# Turkish health system reform from the people’s perspective: a cross sectional study

**DOI:** 10.1186/1472-6963-14-30

**Published:** 2014-01-22

**Authors:** Saad Ahmed Ali Jadoo, Syed Mohamed Aljunid, Seher Nur Sulku, Amrizal Muhammad Nur

**Affiliations:** 1 United Nations University-International Institute for Global Health (UNU-IIGH), Kuala Lumpur, Malaysia; 2 International Centre for Case-Mix and Clinical Coding (ITCC), University Kebangsaan Malaysia Medical Centre, Jalan Yaacob Latiff, Cheras, Kuala Lumpur 56000, Malaysia; 3 Department of Econometrics, Economics and Management Sciences Faculty, Gazi University, Room No: 310 Besevler/, Ankara 06500, Turkey

**Keywords:** Perspectives, Health reform, Turkey

## Abstract

**Background:**

Since 2003, Turkey has implemented major health care reforms to develop easily accessible, high-quality, efficient, and effective healthcare services for the population. The purpose of this study was to bring out opinions of the Turkish people on health system reform process, focusing on several aspects of health system and assessing whether the public prefer the current health system or that provided a decade ago.

**Methods:**

A cross sectional survey study was carried out in Turkey to collect data on people’s opinions on the healthcare reforms. Data was collected via self administered household’s structured questionnaire. A five-point Likert-type scale was used to score the closed comparative statements. Each statement had response categories ranging from (1) “strongly agree” to (5) “strongly disagree.” A total of 482 heads of households (response rate: 71.7%) with the mean age of (46.60 years) were selected using a multi stage sampling technique from seven geographical regions in Turkey from October 2011 to January 2012. Multiple logistic regressions were performed to identify significant contributing factors in this study.

**Results:**

Employing descriptive statistics it is observed that among the respondents, more than two third of the population believes that the changes have had positive effects on the health system. A vast majority of respondents (82.0%) believed that there was an increase in accessibility, 73.7% thought more availability of health resources, 72.6% alleged improved quality of care, and 72.6% believed better attitude of politician/mass media due to the changes in the last 10 years. Indeed, the majority of respondents (77.6%) prefer the current health care system than the past. In multivariate analysis, there was a statistically significant relationship between characteristics and opinions of the respondents. The elderly, married females, perceived themselves healthy and those who believe that people are happier now than 10 years ago have a more positive opinion of the changes. While, the single unemployed from rural region who perceived themselves as unhealthy and believe that people are unhappy now compare to ten years ago showed less positive opinions.

**Conclusions:**

Hence, we conclude that from the people’s perspective overall the health system reforms were most likely successful.

## Background

Turkey is a member of the United Nations (UN), the North Atlantic Treaty Organization (NATO), Group of Twenty (G-20), The World Trade Organization (WTO), The Organization for Economic Co-operation and Development (OECD), and several other regional and international organizations. Its population exceeds 74.5 million. More than two-thirds (67.3%) of its population is within working age group, while the dependent age group constitutes 25.3% and 7.3% in less than 15 years old and 65 years and above, respectively. Annual population growth rate of Turkey in 2011 was 13.5% [1].

Turkish healthcare system was characterized by its highly complex and fragmented provision and financing systems as well as inequalities in access to healthcare. In the year 2003, Turkey started Health Transition Program (HTP) to develop easily accessible, high-quality, efficient, and effective healthcare services for the population, which was also for the sake of pairing its healthcare system with the health regulations of the European Union (EU) and OECD countries [2,3].

This paper aimed to elicit opinions of the Turkish people on health system reform process going on since 2003 using opinion polls survey. The study focused on accessibility, availability of resources, quality of care, information by politicians and mass media, and whether the public prefers the current health system or that provided a decade ago.

### Health transition program (HTP)

Under HTP several measures have been taken: Accessibility: all public health facilities are transferred to be under the authority of the Ministry of Health. This step enables the entire population to access all public health facilities. Financing of healthcare services: the implementation of the “Social Security and Universal Health Insurance Law” in October 2008 extended the insurance coverage to the entire population. The purchaser and provider functions of the Ministry of Health hospitals as well as the insurance organizations were separated. Primary care: family practitioner scheme (FM) was introduced to cover the entire population at the end of 2010. Hospitals: although the administrative and financial autonomy of hospitals was an important component of the reform process, it has advanced at a slower pace. Quality: quality units have been established at the ministerial, provincial, and organizational levels. Efficiency: in 2004, Ministry of Health started a performance-based payment system (P4P) in all its hospitals and healthcare providers, health information system has been upgraded and followed by introduction of a case-mix system, which led to efficient use of resources. Patient’s rights: special units within healthcare institutions that investigate complaints by patients and providers were established as part of the strengthening of patient rights [2,4-7].

### Health indicators

Turkey has achieved remarkable improvements in major health status indicators. Infant mortality rate in Turkey decreased from 31.6 per 1000 live births in 2000 to 10.1 per 1000 live births in 2010, and the maternal mortality rate declined from a national average 19.4 per 100 000 live births in 2008 to 16.4 per 100 000 live births in 2010. Betterment of life expectancy for both men and women increased from 71.0 years in 2000 to 74.3 years in 2010. As the gross domestic product (GDP) increased fourfold between 2000 and 2008, the total expenditure on health as a proportion of GDP also has risen from 4.9% in 2000 to 6.1% in 2008. This is accompanied by increased sharing of health expenditure from public sources as a proportion of total health expenditure from 62.9% in 2000 to 73% in 2008. The share of out of pocket (OOP) payments was 17.4% of total healthcare expenditure in 2008, with a decrease from 27.6% in 2000. The decrease can be mainly attributed to reforms that improved health coverage of the population [2,8-10].

### Responsiveness to patients

An important goal of HTP is to ensure public and patients’ responsiveness [11]. Either individually or combined, three main indicators are used to measure responsiveness: patients’ experience, subjective satisfaction, and subjective expectations with various aspects of care. Literatures indicated that measures of subjective satisfaction or expectations are more difficult to interpret than patients’ experience [12]. However, data needed to compare patient experiences across or between Turkey and other countries is more likely unavailable. The efficiency or the quality of health services cannot be proven on its own. However, any health system that is seeking to obtain good results has to be citizen-oriented and should meet people's expectations [13]. Public participation in the health reform process may be affected positively or negatively with the adoption of the services and thus help get the results much faster. Regular surveys and specifically household surveys to elicit public and patient’s views and experience are increasingly being used as important sources of information on the responsiveness of the health system [12].

### Patients’ satisfaction

Several national and international studies were carried out to assess healthcare system reform in Turkey. However, most of these studies were not designed to find out people's ideas and opinions on the direction of actual reforms in healthcare system. Life Satisfaction Survey, which is periodically conducted by Turkish Statistical Institute (TURKSTAT), reported a growing increase in the proportion of overall satisfaction with health services among Turkish citizens from 39.5% in 2003, just before the beginning of the HTP, to 73.04% in 2010 [14]. Two EUROPEP surveys (Dagdeviren and Akturk [15] & OECD & IBRD/World Bank [8]) investigated satisfaction with primary care services in a large sample of patients spread across 81 Turkish provinces [8,15]. Public satisfaction with most aspects of primary care has increased sharply since the EUROPEP survey reported by Dagdeviren and Akturk [15]. A survey of patients’ expectations about hospital care in Trabzon city found that some of patient’s rights got high evaluation marks such as the right to choose a hospital (85.1%) and the right to receive information about their disease (79.8%), while other rights related to treatment method, privacy statement, and change the health personnel got lower marks [16]. Ankara Doctors Chamber conducted health services satisfaction survey. This study employed outpatients from four hospitals in Turkey. About two-third of the sample was dissatisfied with the current healthcare system [17]. Ali Jadoo et al [7] reported on a survey of level of patients’ satisfaction toward national health insurance in Istanbul city. This small survey included 345 heads of households, who had at least one type of health insurance plan. More than half of the respondents were satisfied with health services that they received [7].

## Methods

### Study population

This was a cross-sectional study conducted in Turkey from October 2011 to January 2012. The sampling method was a self-administered survey of heads of households by using a multistage sampling technique: first, we divided Turkey into seven geographical regions (Aegean, Black Sea, Central Anatolia, Eastern Anatolia, Marmara, Mediterranean, and South-eastern Anatolia); then, we randomly selected one province from each geographical region; two districts from each selected province; two municipalities from each selected district; two quarters from each selected municipality; six blocks from each selected quarter; and two heads of houses from each selected block. Trained interviewers were recruited to explain the objectives and conditions of the study to respondents. Each eligible respondent received one version of the questionnaire during the weekend days and collected back a week later by interviewers. Respondent or a member of the respondent household to be included in the study, must have been under one of the health insurance schemes; used at least two or more types of health care services during the last ten years; was at least 18 years old or older when health reform process began since 2003 and willing to participate. All healthcare providers, health management personnel, politicians, media workers and mentally unstable were excluded. Supervision during data collection phase was ensured in all stages. Out of 672 distributed questionnaires, 482 completed questionnaires were used for analysis, making a response rate of 71.7%.

### Questionnaire

A self-administered modified questionnaire was employed to collect the public opinion. The questionnaire had two parts: sociodemographic items on age, gender, marital status, education, area of residency, happiness, health status, and occupation. For the purpose of statistical analysis, we categorized some of the independents variables into two categories. The second part contained 17 items designed to assess people's opinions about the healthcare reforms. Five aspects were measured: accessibility (five questions), availability of resources (three questions), quality of care (four questions), and opinion regarding the public attention paid to the healthcare reforms by politicians and mass media (three questions). Two questions asked for people’s preferences about the old and the new healthcare system and whether they prefer health insurance coverage now or that available a decade ago.

A five-point Likert-type scale was used to score the closed comparative statements. Each statement had response categories ranging from (1) “strongly agree” to (5) “strongly disagree.” Negatively worded questions were reverse scored (so that 1 = 5, 2 = 4, etc.). For the purposes of cross-tabulation and logistic regression analysis, and to assess the people’s opinion toward health reform process, we needed to effectively dichotomize the number of respondents into two contextual groups: high and low (positive and negative opinion) on each dimension and on overall scale. Therefore, dummy variables for (0) negative and (1) positive opinion were constructed and summed from the seventeen items as originally scored (1–5) (range 17–85). Decision was made to dichotomize the summary score based on a median split (cut-off point) into (0) for low or negative opinion toward health reform process and (1) for high or positive opinion toward health reform process as two dependent variables.

### Ethics

This study was approved by ethics committee of National University of Malaysia- Medical Center (UKMMC)*,* code number (FF-175- 2011). All respondents gave their written informed consent.

### Data analysis

Normality tests were done and all the quantitative data were found to be normally distributed. Data collected were analyzed using Statistical Package for Social Science (SPSS) program version 16.0. Cross-tabulation (Chi-square test) was used for dichotomized characteristics of respondents and people’s opinion. Multiple logistic regressions were performed to identify significant contributing factors for people’s opinions in this study.

## Results

### Respondent’s sociodemographic characteristics

Respondents' age ranged from 28 to 73 years, but the mean age was 46.60 ± 11.85 years. The highest response rates at 52.1% were at the age of 45 years and above. More than half were married (59.8%), with males (51.7%). However, less than half (48.8%) of the respondents had completed their academic education and were considered as highly educated (with university certificate and above) when compared with 51.2% who completed their primary and secondary education (considered as low-level education). More than two-third (68.9%) of the respondents believed that people currently are happier than they were a decade ago. In terms of household occupational status, 36.9% of them worked in the governmental sector, 31.5% in the private sector, and 18.9% were self-employed, while 12.7% were unemployed. About two-third of the participants were from urban regions (63.7%) and considered themselves as healthy (69.1%). Table 1 shows the demographic characteristics of the respondents.

**Table 1:** Frequency distribution of categorized socio-demographic factors of respondents (n = 482)

**Respondents' characteristics**	**Categorized variables**	**Freq.**	**%**
Age	< 45	231	47.9
	≥ 45	251	52.1
Gender	Male	249	51.7
	Female	233	48.3
Marital status	Single	194	40.2
	Married	288	59.8
Education	High Education	235	48.8
	Low Education	247	51.2
Area of residency	Rural	175	36.3
	Urban	307	63.7
Happiness	Unhappy	150	31.1
	Happy	332	68.9
Health Status	Unhealthy	149	30.9
	healthy	333	69.1
Occupation	Unemployed	61	12.7
	Employed	421	87.3

### Opinions on changes in healthcare

More than two-third (69.3%) of the respondents have positive opinions when the current situation is compared with that a decade ago in terms of accessibility, availability of resources, quality of care, and the attitudes of politicians to healthcare. At the same time, 77.6% of the respondents prefer current situation than that in the past. Table 2 shows the overall respondents’ opinion by domains.

**Table 2:** Frequency distribution of respondents’ opinion by five domains (n = 482)

**Domains**			**Positive opinion**	**Negative opinion**
	**Mean (SD)**	**Median**	**Freq. (%)**	**Freq. (%)**
Accessibility	14.06 (2.40)	13	395 (82.0)	87 (18.0)
Availability of resources	11.78 (2.88)	12	361 (74.9)	121 (25.1)
Quality	15.74 (3.32)	16	353 (73.2)	129 (26.8)
Attitude	10.24 (2.13)	10	367 (76.1)	115 (23.9)
Preference	7.77 (1.81)	8	348 (72.2)	134 (27.8)
Overall people view	59.60 (10.25)	61	334 (69.3)	148 (30.7)

A vast majority of respondents (82.0%) believed that there is an increase in accessibility due to the changes in the last 10 years. All questions were positively answered. Respondents agreed or strongly agreed (77.6%) that healthcare is easier to get today when compared with that a decade ago. About 85.4% of the respondents have no difficulty in getting drugs and treatment; in addition, 80.3% of the respondents said that medical treatment is more accessible now for everybody when compared with that available a decade ago. Regarding the payments for medication, only 20.0% believed that there is a higher payment when compared with that a decade ago, while 63.3% of the respondents had a different opinion (with 17.0% undecided). When asked if they had to wait longer for medical treatment now when compared with that 10 years ago, 78.9% of the respondents disagree or strongly disagree, while 7.9 agree or strongly agree. Most of the respondents (73.7%) thought that the resources are available such as enough doctors and enough hospitals when compared with that a decade ago.

Concerning the quality of care during the past 10 years, 72.6% of the respondents showed a positive opinion. Majority of respondents (80.7%) thought that there had been an improvement in quality. Art of care in relation to patients (as component of quality of care) was described by physicians’ attitude and information delivered to the patient. About 80.0% of the respondents considered that physicians are much friendlier and 76.0% of those surveyed felt that doctors gave them more information these days when compared with that 10 years ago; in addition, more than 80.0% of the respondents thought that their doctor’s office has everything needed to provide complete care when compared with that a decade ago.

Regarding the opinion on information by politicians and mass media, most of the respondents (76.1%) had a positive impression. Of the respondents, 73.3% agreed or strongly agreed with the statement that people feel more responsible for their own health when compared with that 10 years ago. Respondents were also asked their opinions about information received these days regarding health risks and health behaviours when compared with that 10 years ago. Of the respondents, 23.1% believed that they are less informed now, while 61.8% believed that they are better informed now (with 15.1% undecided). Also, most of the respondents (75.3%) believed that healthcare gets more attention from politicians now, while 13.7% of the respondents have the opposite opinion.

When asked if they prefer to go back to the healthcare system as it was 10 years ago, the majority of respondents (77.6%) prefer the current healthcare system than that in the past, while 13.4% of the respondents would prefer to live in the past system (with 9.1% undecided). Population was also asked about the current health insurance coverage; more than two-third of the population (76.6%) stated that health insurance coverage right now is better than that a decade ago. Table 3 shows opinions on changes in health care (in %) by domains.

**Table 3:** Opinions on changes in health care (in %)

**Statement on:**	**Strongly disagree**	**Disagree**	**Unsure**	**Agree**	**Strongly agree**
**Accessibility statements**					
Health care is easier to get as compared to a decade ago.	4.4	7.3	10.8	40.0	37.6
Drugs and treatment are more difficult to get than a decade ago.	42.9	42.5	6.0	6.6	1.9
You have to pay more for medical treatment compared with a decade ago.	28.2	34.9	17.0	12.9	7.1
Medical treatment is more accessible now for everybody as compared with a decade ago.	3.1	4.8	11.8	47.3	33.0
Patients have to wait longer for medical treatment now as compared with a decade ago.	43.6	35.3	13.3	5.8	2.1
**Availability of resources statements**					
There are enough doctors in this area as compared to a decade ago.	5.4	8.9	13.5	45.4	26.8
There are enough doctors in the area who specialize as compared to a decade ago.	4.6	7.3	4.4	45.4	38.4
There are enough hospitals in the area as compared to a decade ago.	4.8	9.3	8.1	43.4	34.4
**Quality statements**					
The quality of care improved as compared to a decade ago.	2.9	7.3	8.9	52.1	28.6
Doctors are much friendlier as compared to a decade ago.	4.1	9.1	7.7	52.5	26.6
Doctors give you more information as compared to a decade ago.	3.0	9.8	11.2	52.1	23.9
My doctor’s office has everything needed to provide complete care as compared to a decade ago.	3.7	8.3	6.4	45.9	35.8
**Attitude statements**					
People feel more responsible for their own health as compared with a decade ago.	5.0	7.5	14.1	43.4	29.9
The population is less informed about health risk and healthy behaviour as compared with a decade ago.	25.9	35.9	15.1	15.4	7.7
Health care gets more attention from politicians as compared with a decade ago.	4.4	9.3	11.0	39.8	35.5
**Preference statements**					
I would like it when we could go back to the health care system as it was a decade ago.	28.4	49.2	9.2	9.1	4.1
I prefer health insurance services now than as it was a decade ago.	4.4	7.9	11.2	47.3	29.3

### Opinion by socio-demographic factor

Multivariate analysis indicated that there was a significant relationship between all eight factors and people’s opinion (p value < 0.05). The old age (≥ 45 years) group (p = < 0.001, prevalence odds ratio [POR] = 0.392, 95%CI 0.21-0.74), females, (p = < 0.001, [POR] = 3.395, 95%CI 1.78-6.47), married, (p = < 0.001, [POR] = 3.012, 95%CI 1.62-5.60), have high education, (p = < 0.001, [POR] = 4.639, 95%CI 2.44-8.82), from urban region, (p = < 0.001, [POR] = 5.541, 95%CI 2.99-10.25), who believed that they are happier now than 10 years ago, (p = < 0.001, [POR] = 3.074, 95%CI 1.60-5.91), perceived themselves healthy, (p = < 0.001, [POR] = 2.984, 95%CI 1.57-5.67), and employed, (p = < 0.001, [POR] = 4.176, 95%CI 1.71-5.67) were significant. Upon controlling for confounders (Table 4), only respondents aged ≥ 45 years old, females, married, have high education, from urban region, believed that they are happier now than 10 years ago, perceived themselves healthy and were employed, significantly associated with positive people’s opinion toward health reform process.

**Table 4:** Association between sociodemographic factors and respondents’ opinion (n = 482)

**Factors**	**Positive opinion**	**Negative opinion**	**Wald**	**p-value	Exp(B)^*^[POR]	**95% C.I.**
	**freq. (%)**	**freq. (%)**				
Age						
≥ 45	210 (83.7)	41 (16.3)	50.83	<0.001	0.392	0.21-0.74
< 45	124 (53.7)	107 (46.3)	Referent			
Sex						
Female	207 (88.8)	26 (11.2)	80.99	<0.001	3.395	1.78-6.47
Male	127 (51.0)	122 (49.0)	Referent			
Marital status						
Married	250 (86.8)	38 (13.2)	103.12	<0.001	3.012	1.62-5.60
Single	84 (43.3)	110 (56.7)	Referent			
Education						
High	192 (81.7)	43 (18.3)	33.13	<0.001	4.639	2.44-8.82
Low	142 (57.5)	105 (42.5)	Referent			
Area of residency						
Urban	334 (69.3)	148 (30.7)	102.32	<0.001	5.541	2.99-10.25
Rural	72 (41.1)	103 (58.9)	Referent			
Happiness in 10 years						
Happy	286 (86.1)	46 (13.9)	142.42	<0.001	3.074	1.60-5.91
Unhappy	48 (32.0)	102 (68.0)	Referent			
Health Status						
Healthy	281(84.4)	52 (15.6)	115.32	<0.001	2.984	1.57-5.67
Unhealthy	53 (35.6)	96 (64.4)	Referent			
Employment						
Employed	320 (76.0)	101 (24.0)	70.50	<0.001	4.176	1.71-5.67
Unemployed	14 (23.0)	47 (77.0)	Referent			

*[POR] prevalence odd ratio, ** p-value significant at < 0.05.

## Discussion

Our study brought out opinions of the Turkish people on HTP process employing opinion polls survey. We employed a survey, which questions the subjective satisfaction of people with various aspects of care. In the survey, five aspects of care were measured: accessibility, availability of resources, quality of care and public opinion regarding the attention paid to the healthcare reforms by politicians and mass media, and measuring the preference.

The important specialty of the survey is that questions in the survey do not question people’s opinion about only the current healthcare system but their opinion comparing the current system to the one a decade ago (2003). Therefore, the results of the survey provide comparison of the opinion of the general public about the system before and after the HTP reforms, and measure the success (or failure) of the reforms from people’s perspective.

The overall respondents’ opinion was positive (69.3%) when the current situation was compared with that a decade ago. At the same time, 77.6% of them preferred current situation than that in the past. The results were not surprising, because Turkey has been engaged in health sector reforms since 2003. One of the important goals of the reforms is to establish a healthcare system that is responsive to patients. Before the reforms due to the lack of enough health personnel, there was overcrowding in public hospitals, long waiting times, poor quality, poor responsiveness, and low patient satisfaction with the health system [18]. Performance-based payment system (P4P) and the family medicine (FM) system were among the key interventions to address these problems [8]. The P4P system links the individual bonus payments of the health personnel to their performance and encourages them to provide productive and qualified services [18]. An aggregate amount of bonus payments is adjusted by the institutional performance multiplier, which is given to the MoH hospitals by the MoH according to institutional performance audit results considering equally weighted five topics: a) access to examination rooms, b) hospital infrastructure and service processes, c) patient and caregiver’s satisfaction, d) institutional productivity, and e) institutional targets [19]. Currently, a human-oriented service principle adopted FM system covers the whole country. The main aims of FM system are to provide primary healthcare services to people in need with an easy access to health service utilization and to implement a reasonable referral system that is expected to avoid excessive workload and help to allot adequate time for patients in secondary healthcare level [8,20,21].

In our study when we asked people’s opinion on the availability of health personnel and facilities we observed that 73.7% of them though that were enough doctors and hospitals currently as compared to a decade ago. Actually, the number of hospitals and primary healthcare institutions clearly increased when compared with that before the HTP [9]. The HTP reforms such as the rights to choose physician and type of hospital (including the private one), which has been implemented since 2004, as well as the implementation of P4P that provided good incentives for many specialist doctors who left their private clinic and joined hospitals. However, the number of physicians per hundred thousand had not changed significantly. Turkey ranks at the bottom of the WHO European Region. The fact that in our study most of the participants were from the urban regions (63.7%), where there was the highest proportion of doctors and specialists, when compared with the rural regions may partly explain our findings [9].

Healthcare system reform is rarely evaluated from people’s perspectives in most of the developed and developing countries. This reflects lack of interest of political parties in managing public expectations and preferences. In contrast, the expanded oversight role of modern mass media for criticizing and clarifying health system reform procedures in addition to portraying trends in mass opinion in the last two decades has impact on public preferences, especially among the low and medium politically aware ones [22,23]. Bostan et al. [16] showed that “the level of the expectations of the patient was high on the factor of receiving information” [16]. In fact, health system reform in Turkey has gained special attention from the highest governmental authorities and various media coverage [9,24]. This care has touched simple citizens who expressed positively by three quarters in our study.

Furthermore, we have analyzed people’s view about the new healthcare system when compared with pre-reform system by socio-demographic factors. In multivariate analysis, by employing chi-square tests, we observed a statistically significant relationship between characteristics of respondents and their opinions. We have found that the elderly, married females, those who believe that people are happier now than 10 years ago, and those who live in urban area have more positive opinion on the changes. As commonly found in the literature, older people have a critical opinion. They are the major recipients of healthcare services and their judgment is mostly linked to their experience [7,25,26]. As our analysis found out, the elderly has more positive opinions, it is an important indicator of the success of the HTP process from the people’ perspectives.

Turkish family consists of four persons on the average. In the new legislation, healthcare and medication is free of charge up to eighteen years old. Pregnant women are encouraged to contact maternal healthcare centres with monthly incentives [9], in addition to many special programs for people with special needs, elderly, and chronically ill patients. These services significantly reduced the economic burden on the family [9]. Similarly, in Croatia, older people, women, and those with lower education or lower income have a negative opinion toward the patients’ copayments for various health services [26].

In our study we observed that the unemployed, low educated those who perceived themselves as unhealthy and those who live in rural area showed less positive opinions on the HTP changes. In Turkey, the need for healthcare across regions and social classes is not equally distributed. People who live in rural regions have disadvantaged socioeconomic conditions; usually, they have lower education and income level. The mortality and morbidity rates tend to be significantly higher among lower income. In fact, the inverse care rule (access to care inversely related to need for care) still exists in Turkey even after the HTP reforms since significant differences in the number of health staff remain between the least developed regions and other regions [8,18,27,28].

Although HTP reforms united different public social security schemes under one umbrella, it lags behind in integrating the unemployed or informally employed into system [7,29]. These reasons may explain why unemployed, low educated respondents and those who live in rural area have less positive opinions about the HTP reforms and reported more difficulties in access. Also in Turkey, people with high education level have lower probability of having out of pocket expenditure as they have better health status [7,29]. Thus, people with low education level have higher probability of having out of pocket expenditure as they may have worse health status. Imply that the results support the original hypothesis that low educated people would be less satisfied with the healthcare system.

We also compare our findings with the results of the life satisfaction surveys published periodically by TURKSTAT. TURKSTAT claims that overall satisfaction with health services among Turkish citizens increased from 39.5% in 2003, just before the beginning of the HTP reforms, to 66.5% in 2007 and 75.85% in 2011 [14]. Study done by Ali Jadoo et al. [7] indicated a high level of satisfaction toward national health insurance in Istanbul city [7]. These results are parallel to our findings as we have found that a vast majority (70%) of respondents have positive opinion on current health system when compared with the system a decade ago. Similarly, Romanian healthcare system has undergone a reform process and have been evaluated a decade later by Bara et al. [25], which found that more than 74% of Romanian people preferred the current healthcare system than that a decade before [25]. Balabanova and McKee [30] evaluated the public perspective toward reforming healthcare financing in Bulgaria. They concluded that "people prefer a universal health insurance system that is equitable, transparent and accountable to most stakeholders, as an antidote to the former tax-based model" [30]. Koch [23] noted that people’s opinion on the government-provided health insurance may be changed greatly over a relatively short period of time [23]. In the case of Turkey, although universal health insurance system is a recent one, the results appeared through reduction of the gap between public satisfaction in Turkey and other European countries [9] and through preference of the current health insurance system on the past by 76.6% of our study participants. Although the prominence of arguments advanced by political elites may affect the structure of preferences of the community, it seems that the presence of comprehensive health insurance has a positive impact on people's opinion. In contrast, for example, one-third of the uninsured in United States were dissatisfied with the quality of care they received [31]. The absence of universal healthcare coverage in United States has created serious problems for many Americans who do not have health insurance [32], such as delayed treatment for a serious illness [31-33].

Finally, in 2011, Ankara Doctors Chamber (Ankara Tabip Odası, ATO) conducted a health services satisfaction survey. This survey has been employed on 290 patients who have received outpatient services from four hospitals. The results show that 65% of the patients are not satisfied with the current healthcare system [17]. First of all, ATO’s survey results state the view of the patients to the current system, but it does not elicit views of the Turkish people on health system reform process going on since 2003. Second, this survey was restricted to outpatients only, and third, as in our analysis, unhealthy people have less positive views on healthcare system. Thus, ATO’s result does not represent the general idea of public, but it probability has a sample selection bias.

### Limitation of study

First, despite use of multistage sampling method to collect the data nationwide and the benefit of household survey as a good source of information, we think that to elicit public opinion; the sample has to be broader including all the provinces. Second, the questionnaire was modified with close comparative statements with five-point Likert-type scale; this may be considered a bias as the public have limited place for their expression. Third, we tried to add more healthcare aspects (accessibility, quality of care, availability of resources, attitude of politicians, and media); however, other aspects such as continuity of care have to be included. Fourth, the idea of comparing the health system situation over a long period of time (i.e. 10 years), depending on the patient's memory, may be punctuated by the loss of important events that most probably will affect the assessment of health reform process from patient’s point of view and considered a recall bias.

## Conclusion

The past 10 years in Turkey witnessed a qualitative and quantitative health system reform development. Universal health insurance was on top of many achievements, in addition to improvements in major health status indicators. OECD and the World Bank [8] stated that “the government’s strong commitment and leadership, accompanied by strong economic growth, have resulted in the implementation of long-desired reforms in the health services delivery system” [8]. These changes have affected the citizen's life and made the difference with the past decade through increased accessibility, availability of resources, and quality of care provision.

The democratic governments evaluate their success through the ballot boxes. However, the need to elicit the view of the general public is very important to assess the process of reform. Knowing the opinions of the population about the reforms has become a healthy sign in political life and can help in explaining the possible causes of the unintended consequences of the reform process [34]. Thus, in our study we aimed to elicit opinion of the Turkish people on health system reform process going on since 2003, focusing on several aspects of health system by conducting people’s opinion surveys. Depending on our finding from the opinion polls analyses we conclude that from the people’s perspective overall the health system reforms were most likely successful in Turkey. We finally recommended further research about the health reform process in Turkey as well as the reliability and validity of the questionnaires used to elicit the public point of view regarding this issue.

## Abbreviations

UN: United Nation; NATO: The North Atlantic Treaty Organization; G-20: Group of twenty; WTO: The World Trade Organization; OECD: The Organization for Economic Co-operation and Development; EU: European Union; HTP: Health Transition Programme; FM: Family Practitioner Scheme; P4P: Performance-based payment system; GDP: Gross domestic product; OOP: Out of pocket; TURKSTAT: Turkish Statistical Institute; SPSS: Statistical Package for Social Science (SPSS); ATO: Ankara Tabip Odası, Ankara Doctors Chamber.

## Competing interests

The authors declare that they have no competing interests.

## Authors’ contributions

SAAJ: conceived study, collected, coded and analyzed the data, and wrote the first and final draft of the article. SA: advised and contributed to the study design and data analysis. SNS: advised and contributed to the data analysis and writing of article. AMN: advised in the study design. All authors have read and approved the final manuscript.

## Pre-publication history

The pre-publication history for this paper can be accessed here:

http://www.biomedcentral.com/1472-6963/14/30/prepub
